# Particulate matter air pollution: effects on the respiratory system

**DOI:** 10.1172/JCI194312

**Published:** 2025-09-02

**Authors:** Robert B. Hamanaka, Gökhan M. Mutlu

**Affiliations:** Department of Medicine, Section of Pulmonary and Critical Care Medicine, The University of Chicago, Chicago, Illinois, USA.

## Abstract

Air pollution comprises a complex mixture of gaseous and particulate components. Particulate matter (PM) air pollution is associated with 4.7 million premature deaths per year. Among modifiable risk factors, air pollution exposure contributes to 8% of disability adjusted life years and ranks above factors such as high blood pressure, smoking, and high fasting plasma glucose. As the site of entry, exposure to PM air pollution causes respiratory symptoms and is a significant cause of respiratory morbidity and mortality. In this Review, we discuss the studies that link air pollution exposure with respiratory diseases. We review the epidemiological evidence linking PM exposure and lung diseases including asthma, chronic obstructive pulmonary disease, pulmonary fibrosis, pneumonia, acute respiratory distress syndrome, and lung cancer. We also provide an overview of current knowledge about the mechanisms by which PM exerts its biological effects leading to adverse health effects in the respiratory system.

Air pollution is composed of a complex mixture of particulate and gaseous components. Toxicological and epidemiological study of air pollutants began in earnest in the mid-20th century after industrial smog events, including the Great Smog of London of 1952, caused acute rises in hospitalizations and deaths. These studies have consistently shown that ambient air pollution is the largest environmental health risk factor, contributing to as many as 4.7 million premature deaths per year (1). Among modifiable risk factors, air pollution exposure contributes to 8% of disability adjusted life years, ranking above factors such as high blood pressure, smoking, and high fasting plasma glucose (2). Most of the excess deaths linked to air pollution are due to acute ischemic/thrombotic cardiovascular events (3); however, chronic air pollution exposure is also a significant cause of respiratory morbidity and mortality. In this Review, we discuss the studies that link air pollution exposure with respiratory diseases in humans.

## Components of air pollution

**Figure 1:**
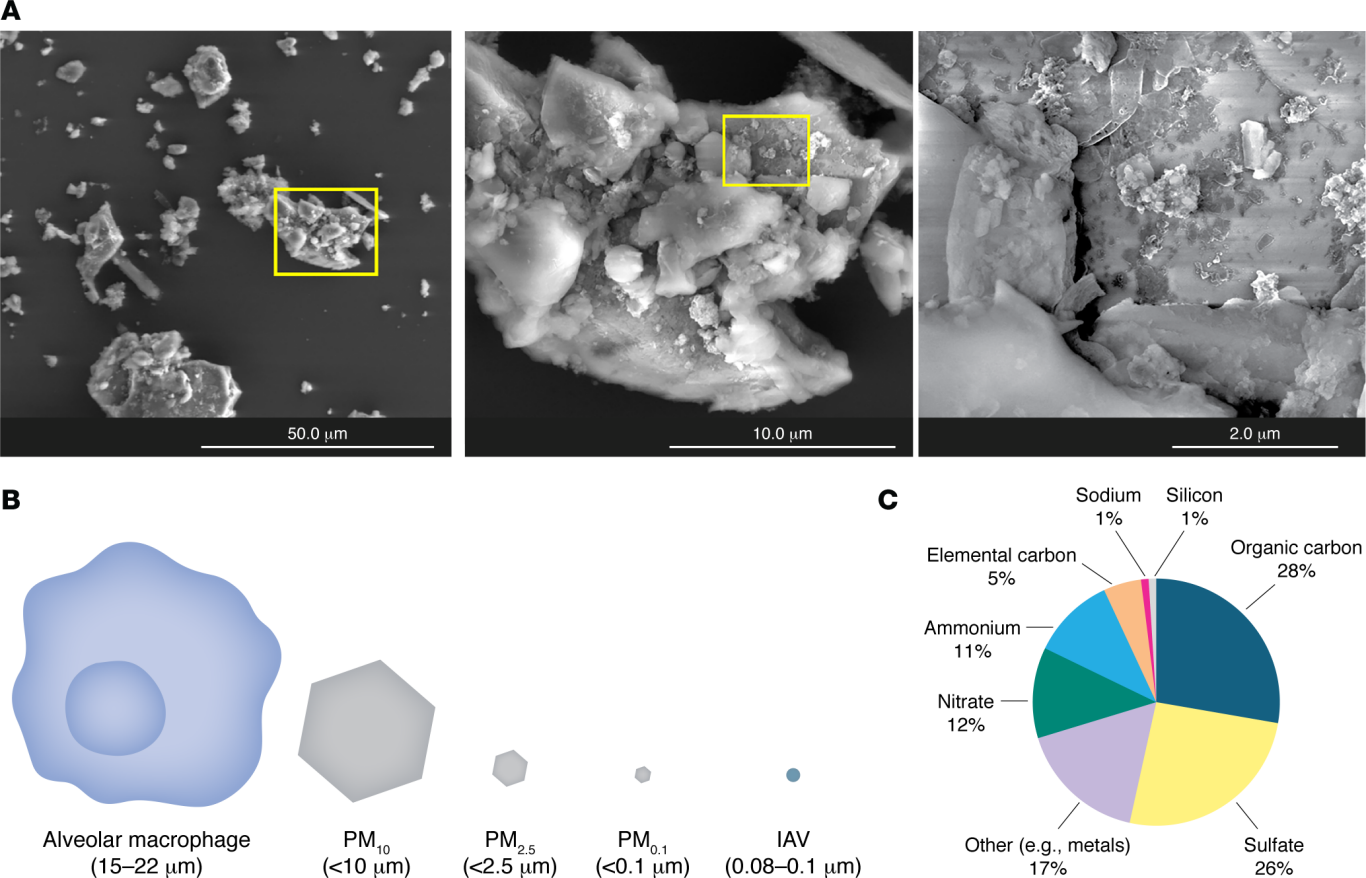
Particulate matter size and components. (**A**) Images of urban particulate matter (PM) generated by scanning electron microscopy. Original magnification, ×2,000 (left), ×10,000 (center), and ×50,000 (right). (**B**) Relative sizes of PM_10_ (coarse), PM_2.5_ (fine), and PM_0.1_ (ultrafine or nanoparticles) in comparison to alveolar macrophage and influenza A virus (IAV). (**C**) Common components of PM. Other category includes metals (Al, K, Ca), transition metals (Fe, Zn, Cd, Ti, Ag, Cu, Mn, Au, Mg, Hg, Cr, Zr, Ni, V, and Co), nonmetals, halogens, and lanthanides (7, 272).

The components of air pollution vary greatly depending on the source of production, emission rate, and weather conditions. Gaseous components of air pollution include sulfur dioxide (SO_2_), nitrogen dioxide (NO_2_), nitric oxide (NO), ozone (O_3_), and carbon monoxide (CO) (4, 5). The particulate component of air pollution consists of carbon-based particles onto which various metals and organic chemicals are adsorbed. Common components of particulate matter (PM) are elemental carbon and organic carbon molecules, such as polyaromatic hydrocarbons (PAHs), nitrates, sulfates, sodium ion, and silicon as well as metals, including transition metals (e.g., Cd, Co, Cr, Fe, Ni, vanadium [V]) (6, 7) (Figure 1). PM is categorized based on particle size. Coarse particles with a diameter of less than 10 μm (PM_10_) are derived from both natural and industrial sources. These particles do not generally penetrate beyond the upper airway. Fine particles have a diameter of less than 2.5 μm (PM_2.5_), and ultrafine particles (nanoparticles) are those that are less than 0.1 μm diameter (PM_0.1_). They are produced by combustion of fossil fuels and penetrate into the small airways and alveoli (8, 9). This makes PM_2.5_ and PM_0.1_ greater threats to respiratory health than PM_10_.

PM and NO_2_ (along with O_3_, which is a secondary pollutant) are the main industrial and traffic-related air pollutants measured in the industrialized world. SO_2_ is produced from combustion of high sulfur-containing fuels such as coal, and its levels have decreased in most of the world (6, 10). Indoor production of air pollutants through cooking and heating are also major sources of exposure-related health effects, particularly in the developing world, where burning of biomass is a major source of energy (11). Due to the coproduction of air pollutants, it is often difficult to determine the independent contribution of each pollutant with health effects; however, levels of PM_2.5_ have consistently been correlated negatively with cardiovascular and respiratory outcomes. Under the Clean Air Act, the US Environmental Protection Agency regulates PM_2.5_ and PM_10_ levels to establish National Ambient Air Quality Standards to protect public health and the environment (12) (Table 1).

## Epidemiology

### Time-series studies.

The Great Smog of London directly contributed to an estimated 12,000 deaths, with tens of thousands more suffering adverse health effects afterward (13, 14). Similar observations linked with industrial smog events in Europe and the United States also provided evidence for the adverse effects of air pollution exposure on health; however, it was not until the late 20th century that a greater understanding of confounding factors such as weather, temperature, and lag effects as well as advances in statistical analysis allowed for epidemiological associations between air pollution and health outcomes to become apparent.

Large, multicity epidemiological studies, including the National Morbidity, Mortality, and Air Pollution Study (NMMAPS) and Air Pollution and Health: A European Approach (APHEA) studies, showed that increases in PM and other pollutants were associated with significant increases in all-cause mortality (15–19). Increased hospitalizations from cardiovascular and respiratory events were also associated with increases in PM levels (20, 21).

The recent Multi-City Multi-Country (MCC) study collected data from over 600 cities, mainly in North America, Europe, and Eastern Asia, and reported similar findings to those of the NMMAPS and APHEA studies, associating PM_2.5_ concentrations with total, cardiovascular, and respiratory mortality (22, 23). This study showed that associations between mortality and PM concentrations were strongest in locations with lower average annual PM concentrations. This finding is consistent with previous studies that showed a biphasic relationship between PM and mortality in which a steep concentration-response relationship is observed at lower PM concentrations, while the curve flattened at higher concentrations (24–26). This flattening of the curve is interpreted to be due to the higher basal PM levels seen in more polluted cities; however, other factors such as younger populations in developing countries may also play a role. The MCC study also confirmed the findings of the NMMAPS and APHEA studies that there is no threshold value of PM pollution below which positive associations are not detectable between exposure and deaths.

### Cohort studies.

The repeated finding that there exists no threshold below which air pollution levels are considered “safe” has led to three recent long-term studies in areas of low ambient pollution in the United States (US Medicare Study) (27), Canada (Mortality-Air Pollution Associations in Low-Exposure Environments [MAPLE] Study) (28), and Europe (Effects of Low-Level Air Pollution: A Study in Europe [ELAPSE Study]) (29), which use large administrative cohorts and advanced exposure assessment techniques to determine the effects of air pollution on health below current air quality standards. All three studies found associations between PM_2.5_ levels and mortality down to the lowest observed PM_2.5_ level of 4 μg/m^3^ (30). Significant correlations between NO_2_ and PM_2.5_ levels were observed with respiratory-related mortality at levels below the current WHO guideline values for both pollutants (29, 31). The findings from these new large studies mostly corroborate the results from older studies using smaller cohorts, including the Harvard Six Cities Study, the American Cancer Association Cancer Prevention Study, and European Study of Cohorts for Air Pollution Effects (ESCAPE) (32–34).

### Interventional and other studies.

The implementation of the Clean Air Act in 1970 resulted in a progressive decline in air pollution levels in the United States. While the decline has occurred gradually over an extended period of time, extended analysis of the NMMAPS and Harvard Six Cities Study revealed that reductions in PM_2.5_ levels contributed to significant increases in life expectancy (35–37). More drastic changes in air pollution occurred in China after the implementation of the Air Pollution Prevention and Control Action Plan (APPCAP) in 2013. Between 2013 and 2017, annual average PM_2.5_ concentrations decreased by one-third, leading to an estimated 47,000 fewer deaths in the 47 cities studied (38).

Recent years have offered several other natural experiments with which the association of abrupt changes in air pollution with health effects can be measured. Before and during the 2008 Olympic Games, Chinese government implemented emission control policies in Beijing and the surrounding area that reduced particulate and gaseous pollutants, including an average 31% decrease in PM_2.5_ (39). This was associated with reductions in pulmonary and systemic markers of inflammation in study participants (40–42) as well as reductions in emergency room visits for cardiovascular and asthma-related events and a decrease in cardiovascular mortality (42–47).

The COVID-19 pandemic led to wide-spread societal shutdowns to limit transmission of the SARS-CoV-2 virus. These shutdowns led to a 31% reduction in PM_2.5_ levels measured across 34 countries during the shutdown periods (48). Early studies have shown that during the time of these policies, significant reductions in non–COVID-19–associated mortality were detected, to which reductions in traffic accidents and exposure to air pollution are proposed as causal (49, 50). Air pollution monitoring and epidemiological modeling allowed for the calculation of the avoided mortality due to improvements in air quality. Reductions in premature mortality were particularly strong in China, which had the most stringent COVID-19 containment policies (51–53).

Exposure to wildfire smoke is a growing public health concern, with pollution due to wildfires increasingly influencing average annual PM_2.5_ concentrations in the United States (54). Health effects of wildfire smoke on cardiovascular and respiratory-related deaths are similar to those seen with industry- and traffic-related pollution (55, 56). Increases in hospital visits and admissions for cardiovascular and respiratory diseases have been seen in Southern California during increased wildfire activity (57–59). Wildfire smoke exposure in California is estimated to have caused over 50,000 premature deaths between 2008 and 2018 (60). Increased asthma- and cardiopulmonary-related hospital visits were also found after wildfire smoke from Quebec spread throughout the northeastern United States in June 2023 (61–63).

The destruction of the New York World Trade Center on September 11, 2001, resulted in an atmospheric dust plume containing thousands of tons of PM from pulverized building materials and combustion products of the fires (64). Many first responders experienced persistent cough accompanied by respiratory symptoms that required medical leave for at least four weeks (65, 66). Extended analysis of lung function from first responders showed an acute decline in lung function (measured by forced expiratory volume in 1 second [FEV_1_]) that did not recover even after years of follow-up (67, 68). Asthma, persistent airway hyperreactivity, obstructive airway disease, and interstitial lung disease have been associated with exposure to the World Trade Center dust (69–72).

## Exposure to air pollution and respiratory disease

Strong associations exist between air pollution exposure and total mortality as well as cardiovascular and respiratory mortality. The MCC study showed that each 10 μg/m^3^ increase in PM concentration was associated with a 0.47% (95% CI, 0.35%–0.58%) rise in respiratory mortality (22). Using International Classification of Diseases (ICD) codes, the ELAPSE project studied four outcomes: nonaccidental, cardiovascular, nonmalignant respiratory, and lung cancer mortality. Significant associations were found between levels of PM_2.5_ and all four mortality outcomes, with lung cancer mortality showing the strongest relationship (29). Follow-up studies showed that PM_2.5_ levels significantly correlated with incidence of asthma, COPD, and lung cancer (73–75). The US Medicare Study, which did not stratify mortality based on cause, found that all-cause and respiratory hospital admissions were significantly correlated with exposure to PM_2.5_ (76). Below we discuss the epidemiologic and experimental evidence for the associations of air pollution exposure with lung diseases.

### Asthma.

Early experiments exposing humans to SO_2_ showed that patients with asthma have greater sensitivity to pollutant exposure than individuals acting as controls (77–79). Subsequent studies have consistently shown that the average local levels of air pollutants, including PM_2.5_, strongly correlate with severity of asthma symptoms and medication usage (80–83), emergency department visits (84–87), and hospitalizations (88–90). A classic example of these observations centers around a labor dispute that led to the closing of a steel mill in Utah Valley for one year over 1986 and 1987. This led to an over 50% reduction in the mean daily high PM_10_ levels. Inpatient admissions to local hospitals for children with bronchitis or asthma similarly halved during the period that the mill was closed (91, 92).

It is also increasingly accepted that air pollution exposure causes new-onset asthma, with evidence being particularly strong for childhood exposure. Birth cohort studies that track residential air pollution exposure have consistently shown that local air pollution, particularly PM_2.5_ and NO_2_ levels, correlate with development of childhood asthma (93–96). The Southern California Children’s Health Study found that children who live in high pollution areas, particularly near major roadways, show reduced lung function (as measured by FEV_1_, forced vital capacity [FVC], and maximum mid-expiratory flow rate [MMEF]) and higher incidence of asthma compared with children from less-polluted areas (97–101). Individuals who moved during the course of the study to areas of lower pollution showed increased rate of MMEF growth compared with participants who did not move (102). As air quality increased during the course of the study, improvements in lung function measurements and reduced incidence of asthma were noted (103, 104).

### COPD.

As with asthma, patients with COPD suffer increases in disease-related health events after increases in local air pollution. Daily gaseous and particulate pollutant levels correlate with reduced respiratory function (FEV_1_ and FVC) and recorded respiratory symptoms and medication usage by patients with COPD (105–108). Emergency room visits, hospital admissions, and mortality due to COPD are positively correlated with elevations in daily particulate and gaseous pollutants (109–114). Patients with COPD are also more susceptible to mortality after local increases in PM_2.5_ than the general population (115).

While tobacco smoking is the greatest risk factor for COPD, increasing evidence suggests that air pollution exposure is also a risk factor. Two recent large cross-sectional studies have shown that local PM_2.5_ levels correlate significantly with the incidence of COPD (116, 117). A large longitudinal study showed that each 5 μg/m^3^ increase in 2-year average PM_2.5_ level was associated with a decrease of 1.18% in FVC and 1.46% decrease in FEV_1_. Compared with the participants exposed to the lowest PM_2.5_ levels, patients with the highest exposure had a hazard ratio of 1.39 (95% CI, 1.16–1.46) for COPD development (118).

### Pneumonia.

Correlations of air pollution exposure with respiratory infections have been observed for nearly 100 years (119). Recent estimates suggest that every 10 μg/m^3^ increase in PM_2.5_ is associated with a 5.4% increase in respiratory tract infections in the Medicare population (120). Children also show heightened sensitivity to PM air pollution, which significantly correlates with pneumonia incidence (121).

Influenza infection is the best studied cause of pollution-associated pneumonia. Multiple studies have shown correlations between PM_2.5_ levels as well as wildfire smoke and incidence of influenza infection (122–125). Correlations of PM_2.5_ with respiratory syncytial virus and SARS-CoV2 infections have also been reported (125–128). While bacterial pneumonias are not as commonly diagnosed as viral pneumonias, mycoplasma pneumonia as well as tuberculosis have also been positively correlated with exposure to PM_2.5_ (129, 130), suggesting that the effects of air pollution exposure on pneumonia are not limited to viral infections.

### Acute respiratory distress syndrome.

Although acute respiratory distress syndrome (ARDS) has heterogeneous causes, including pneumonia, sepsis, trauma, or aspiration, downstream common pathways include inflammatory responses, immune infiltration into the lung, increased endothelial and epithelial permeability, and dysregulated coagulation (131, 132). Increasing findings suggest that air pollution exposure increases the likelihood of developing ARDS. A cohort study of critically ill patients stratified by air pollution exposures at their residences showed that the 3-year average exposure to PM_2.5_, is significantly associated with development of ARDS (133). Exposure to PM_2.5_ was also linked with an increased 90-day mortality rate from ARDS (134).

SARS-CoV-2 infection during the COVID-19 pandemic was a significant cause of ARDS. Several studies have shown correlations between residential exposure to air pollution and severity of SARS-CoV-2 infection and mortality (135–138). Annual PM_2.5_ exposure most closely correlated with COVID-19 hospitalization and death. Similar findings were found when exposure to wildfire smoke was correlated with COVID-19 cases and deaths (139).

### Pulmonary fibrosis.

Compared with other lung diseases, the link between pulmonary fibrosis (PF) and air pollution exposure has only recently been uncovered. The first linkages between PF and air pollution were associations between acute exacerbations and increases in NO_2_ and O_3_ levels (140). Patients with PF have since been shown to exhibit lower lung function (FVC) (141), increased rate of FVC decline (142), and increased rates of mortality associated with exposure level to PM_2.5_ (143). Although these were relatively small studies, a recent larger prospective cohort study showed an inverse correlation between PM_2.5_ exposure and transplant-free survival. Patients with higher PM_2.5_ exposure also had a lower baseline FVC and more rapid FVC decline (144). Another large retrospective study showed that daily hospital admissions based on ICD codes for PF correlated with PM_2.5_ levels, both on the day of admission and average levels in the preceding 4 days (145). There was no correlation when analysis was performed on average pollutant levels for the preceding 30 days, providing evidence of the acute impairment caused by pollution in patients with PF.

More recently, evidence has emerged that incidence of PF also correlates with chronic exposure to air pollution. Each interquartile range increase in PM_2.5_ at residential addresses of UK Biobank participants was found to correlate with a hazard ratio of 1.09 (95% CI, 1.02–1.17) for incidence of PF (146). High attenuation area (HAA) and interstitial lung abnormalities (ILA) are chest tomography-based measures used to identify subclinical forms of interstitial lung diseases and PF. Exposure to PM correlated with the progression of HAA in a prospective cohort enrolled in the Multi-Ethnic Study of Atherosclerosis (MESA) (147). Elemental carbon (a component of PM) exposure correlated with both ILA incidence and progression in participants of the Framingham Heart Study (148). These findings suggest that air pollution exposure may promote the earliest stages of subclinical interstitial and fibrotic lung diseases.

### Lung cancer.

Lung cancer is leading cause of cancer-associated death, and while smoking is the primary risk factor for lung cancer, one-third of lung cancer occurs in nonsmokers, making lung cancer in nonsmokers the fifth leading cause of cancer-associated death (149). The connection between air pollution exposure and lung cancer has been acknowledged since the 1950s (150), and large epidemiological studies have correlated exposure to multiple pollutants, particularly PM_2.5_, with both lung cancer incidence (75, 151, 152) and mortality (29, 153, 154).

Air pollution exposure has also been shown to decrease survival after lung cancer diagnosis. After adjustment for demographic factors, tumor characteristics at diagnosis, and treatment, patients with early-stage tumors living with low PM_2.5_ exposure (<10 μg/m^3^) had a median survival of 5.7 years compared with 2.4 years for patients with high PM_2.5_ exposure (>16 μg/m^3^) (155). Survival after lobectomy was also shown to be reduced by high exposure to PM_2.5_ (156).

### Lung transplant.

As would be expected from the negative effects on pollution on PF and COPD outcomes, annual average PM_2.5_ levels at the residential addresses of lung transplant candidates are associated with increased rates of removal from transplant waiting lists either due to mortality or from clinical deterioration of the patient (157). Air pollution exposure is also correlated with negative outcomes after lung transplantation. Posttransplant development of bronchiolitis obliterans or chronic lung allograft dysfunctions are associated with residential traffic density and proximity to a major roadway (158–160). Transplant recipients with higher PM_2.5_ exposure exhibited reduced FVC and FEV1 and increased rates of graft failure or death (161–163).

## Biologic mechanisms

**Figure 2:**
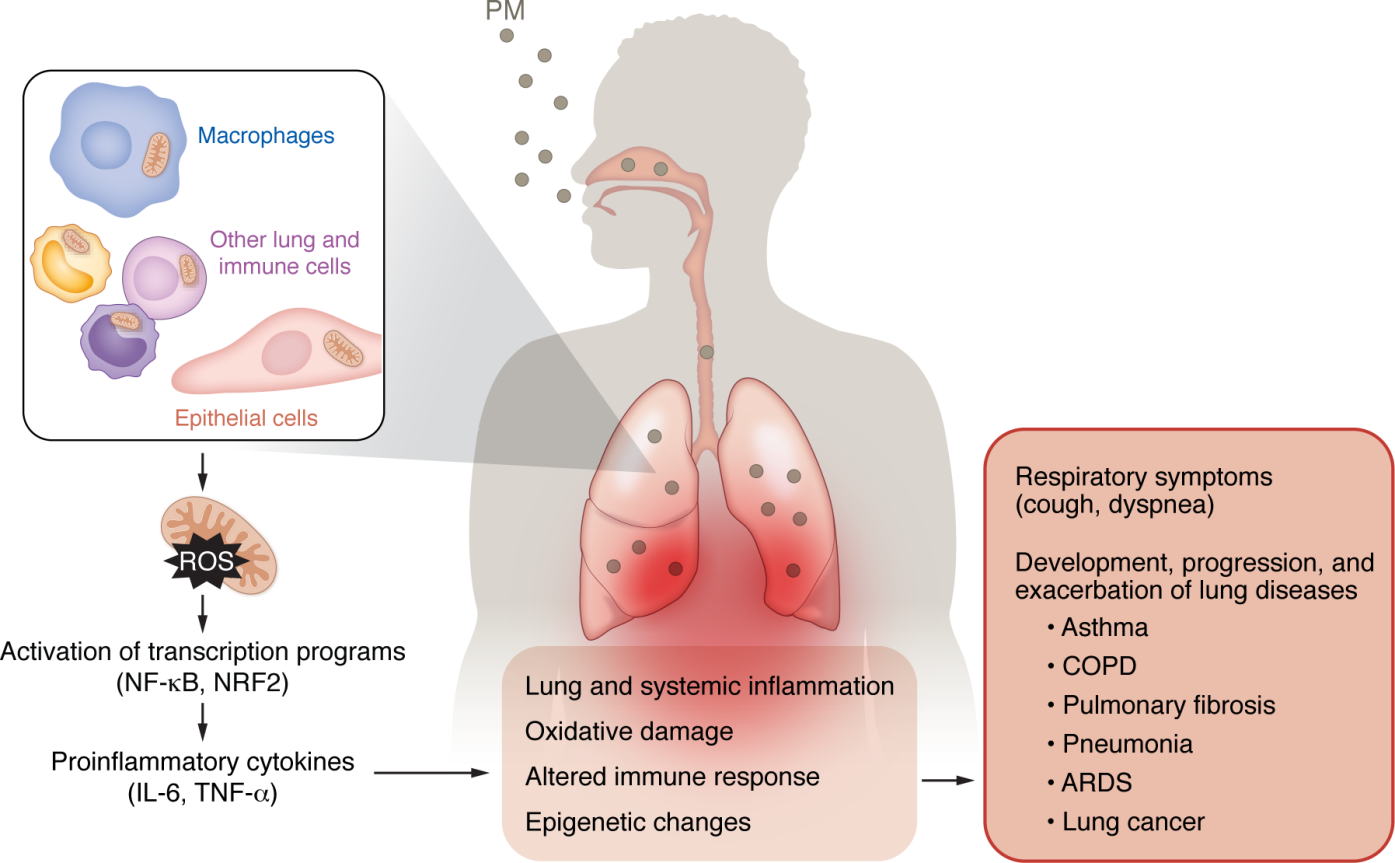
Mechanisms by which PM air pollution affects the respiratory system. Inhaled PM induces an inflammatory response in the lung. PM acts on the cells of the lung, including airway and alveolar epithelial cells and macrophages, causing mitochondrial ROS-dependent transcriptional responses, including NF-κB and NRF2 activation. Oxidative stress promotes production of proinflammatory cytokines and oxidative damage. These PM-induced changes cause lung and systemic inflammation (due to spillover of cytokines into the circulation), altered immune response, and epigenetic changes. Clinically, PM-induced effects are exhibited through the development of respiratory symptoms such as cough and dyspnea in healthy individuals. PM exposure is associated with the development, progression, and exacerbation of lung diseases, including asthma, COPD, pulmonary fibrosis, pneumonia, ARDS, and lung cancer.

While the epidemiological evidence for the effect of PM exposure on lung health is increasingly clear, insight into the mechanisms by which PM exerts its harmful effects is also increasingly understood (Figure 2). Experimental systems have been designed to allow study of both in vivo and in vitro effects of exposure to air pollution particulates. Ambient fine particle concentrators enrich ambient PM_2.5_ from the local air, allowing for inhalation of concentrated ambient particles (CAPs) by humans or animal test subjects (164). Particles can also be collected on filters and used to treat cultured cells in vitro or can be suspended and instilled into the lungs of test animals when inhalation systems are unavailable. Standard reference materials (SRMs), such as SRM1649 Urban Dust and SRM2786, which are commercially available, and certified by the National Institute of Standards and Technology, allow for in vitro exposures of particles that are uniform across laboratories (165). Below, we discuss the current understanding of the mechanisms by which PM exposure leads to lung disease.

### Oxidative stress and inflammation.

It is widely understood that air pollution exerts many of its biologic effects by causing oxidative stress, which promotes subsequent inflammatory responses. Metals contained in PM are capable of redox cycling, and other chemicals adsorbed onto particles, including PAHs, can generate redox-active quinones. NO_2_ and O_3_ are reactive and lead to free radical accumulation (166, 167). Increases in ambient PM_2.5_ or controlled exposure to CAPs are associated with increased levels of DNA and lipid oxidation products in blood, urine, and breath condensate (40, 168, 169). These markers correlate with inflammatory markers such as IL-1β, IL-6, GM-CSF, TNF-α, fibrinogen, and C-reactive protein (170–173).

At the cellular level, in vitro exposure of lung cells, including nasal, airway, and lung epithelial cells, macrophages, and endothelial cells to particles isolated either from ambient air or from diesel exhaust leads to elevations in cellular reactive oxygen species (ROS) levels (174–182). This stress environment activates transcriptional programs, including those regulated by the oxidative stress–responsive transcription factor NRF2 and the proinflammatory transcription factor NF-κB (178, 183, 184). Oxidative stress is required for the biologic effects of PM exposure, as genetic inhibition of oxidant production or treatment of cells with antioxidants is sufficient to inhibit inflammatory cytokine production and proapoptotic signaling (174, 175, 185–188). NRF2-deficient mice exhibited greater lung inflammation after a 24-week inhalation exposure to CAPs, demonstrating the key role that oxidative stress plays in air pollution–induced inflammation (189).

The oxidative and inflammatory environment caused by exposure to air pollution likely plays an important role in the development of inflammatory airway diseases. Markers of oxidative stress are increased in the blood, urine, and breath condensate of patients with asthma and COPD (190, 191). Air pollution–linked cytokines such as IL-1β, IL-6, and TNF-α are highly elevated in the lungs of patients with COPD and play roles in modifying asthma phenotypes (192, 193). Furthermore, both diseases are associated with gene polymorphisms related to oxidative stress phenotypes, suggesting that an individual’s sensitivity to oxidative stress may regulate susceptibility to airway disease (194, 195).

Increased lung inflammation may also be an important cancer-promoting mechanism of air pollution. In a recent study, tumor formation in EGFR- or KRAS-mutant mice was accelerated by intratracheal instillation of exposure to PM (SRM2786) for 3 weeks (196). Despite this difference in tumor burden, there was no significant increase in mutational burden in the PM-exposed mice. The investigators demonstrated that production of IL-1β by lung macrophages promotes expansion of mutant cells and that inhibition of IL-1β using neutralizing antibodies prevented PM-promoted tumor formation. In nonmalignant human lung tissue from two separate clinical cohorts, EGFR and KRAS mutations were present in 18% and 53% of samples, respectively (196). Thus, these findings suggest that the primary mechanism by which air pollution exposure promotes cancer is through inflammatory effects on cells with preexisting mutations. Indeed, a study of UK Biobank participants in which single nucleotide polymorphism data were analyzed alongside air pollution exposure data showed an additive interaction between genetic risk factors and air pollution exposure (197). High air pollution exposure, particularly to PM_2.5_, increased the risk of developing lung cancer in all participants, with patients with high genetic risk scores and high pollution exposure at the greatest risk for lung cancer.

### Mitochondrial dysfunction.

While air pollution gases and PM-adsorbed chemicals and metals can generate free radicals, increasing evidence points to changes in mitochondrial function and mitochondrial production of ROS playing key roles in the response to air pollution. Air pollution particles have been shown to accumulate in the mitochondria of cultured airway epithelial and macrophages, leading to changes in mitochondrial morphology and increased oxidative stress (179, 198–203). Lung cells, including alveolar macrophages (AMs) and epithelial cells, that have been genetically engineered to be deficient in mitochondrial ROS production, or treated with mitochondria-targeted antioxidants or electron transport chain inhibitors, exhibit attenuated inflammatory responses to particles in culture (175, 186, 203, 204).

Similar to air pollution particulates, exposure of cells to cigarette smoke (CS) extract display altered mitochondrial function and increased ROS production (205–207). These mitochondrial changes are also observed in airway epithelial cells from CS-exposed mice and from patients with COPD (207–209). Alterations in mitochondrial turnover may play a role in linking mitochondrial dysfunction with lung phenotypes, as the mitophagy regulator PINK1 was found to be highly expressed in lung tissue of patients with COPD. Furthermore, PINK1-deficient mice were protected against mitochondrial dysfunction, defects in mucociliary clearance, and airspace enlargement after CS exposure (207). Other evidence points to increased mitochondrial iron uptake promoting CS-induced mitochondrial dysfunction, as mice deficient for iron-responsive element-binding protein 2 (IRP2) were also protected from mitochondrial and airway dysfunction after CS inhalation exposure. Supporting the translatability of this finding, *IREB2*, the gene encoding IRP2, has been identified as a COPD susceptibility gene in humans (206, 210). How these mitochondrial regulators affect the response to air pollutants remains to be determined.

### Epithelial dysfunction and senescence.

The lung epithelium is the primary contact for inhaled pathogens and toxins and, thus, plays an important role in barrier function, pathogen clearance, and innate immunity (211). Specialized epithelial populations line the respiratory tract that contribute not only to gas exchange, but also to mucus production and removal of pathogens by mucociliary clearance. Homeostasis of the lung epithelium is regulated by region-specific regenerative programs that restore homeostasis after injury (212). Dysregulation of the immune and regenerative functions of the lung epithelium can contribute to disease (213).

Impaired barrier function has been proposed as a mechanism by which environmental exposures promote allergic diseases, including asthma (214). Exposure to ambient particles disrupts barrier integrity in cultured airway epithelial cells through downregulation of tight junction protein expression (215–218). Increasing cellular antioxidant capacity reduces inflammatory gene expression and prevents barrier loss, suggesting a link between oxidative stress, inflammation, and barrier integrity (215–217). Similar reductions in tight junction function have been observed in vivo (219), and mice exposed to intranasal or intratracheal particles from ambient air or diesel exhaust exhibited significantly elevated allergic responses to subsequent allergen exposure, including eosinophil infiltration, mucus metaplasia, and sneezing (219–221).

Mucociliary clearance is another major defense mechanism affected by air pollution. Studies in rabbits and rats have shown mucous metaplasia and ciliary abnormalities after exposure to either CAPs or wood smoke (222, 223). This is consistent with in vitro studies on human airway epithelial cultures, which have shown that particles from ambient air or diesel exhaust increase expression of mucus secretion genes while ciliary genes and beat frequency are reduced (224, 225). Impaired mucociliary function can promote respiratory infections and is implicated in the pathogenesis of COPD (226, 227).

Senescence affects the ability of the lung to regenerate after injury and is a hallmark of lung fibrosis (228, 229). Exposure to ambient particles induces senescence in cultured lung epithelial cells and fibroblasts (230, 231). Moreover, growth of alveolar organoids was impaired after exposure to diesel exhaust particles (232). While effects of air pollution exposure on lung senescence have yet to be demonstrated in vivo, PM_2.5_ and black carbon exposure have been shown to be inversely correlated with telomere length in circulating blood cells (233–235). Thus, it is likely that air pollution exposure affects senescence and regenerative responses in the lung.

Inflammatory responses to air pollution may also impair lung regeneration and promote fibrosis. Epithelial injury in the lung leads to development of a keratin 5–expressing, migratory, “fluid” epithelial phenotype that promotes wound closure (236). Dysregulation and persistence of this fluid phenotype is found in PF and was shown to be promoted by IL-6 (237). Lung and circulating IL-6 levels increased in mice after exposure to CAPs, and IL-6 plasma levels correlated with PM exposure in humans (171, 173, 186, 238), potentially providing a link between exposure, epithelial remodeling, and lung fibrosis.

### Altered immune response.

Air pollution exposure also affects how the immune populations of the lung respond to inhaled pathogens. AMs respond to PM exposure with upregulation of inflammatory cytokines including IL-1β, IL-6, IL-8, TNF-α, and GM-CSF (171, 239). Elimination of AMs in mice prevented pulmonary and systemic increases in IL-6 and TNF-α after either inhalation of CAPs or intranasal instillation of ambient particles (238, 240). However, long-term exposure to particles by intranasal instillation has been shown to decrease the ability of AMs to secrete IL-6 and IL-1β, resulting in increased death in mice subsequently exposed to influenza (241). In vitro cytokine induction (including IL-6, IFN-β, IL-1β, and TNF-α) after lipopolysaccharide or virus exposure was also blunted by previous exposure to particles from ambient air or diesel exhaust (242–244). Moreover, the ability of macrophages to conduct phagocytosis was impaired by CAP particle exposure (245). These changes suggest that PM causes a state of immune insensitivity, which may predispose to pulmonary infections.

PM exposure also affects lymphocyte populations in the lung. Dendritic cells exposed to ambient particles promote naive CD4^+^ T cell proliferation but with a reduced proportion of Th1 effectors (246). A similar reduction in Th1 cells was observed after in vivo inhalation exposure to particles, which correlated with more severe influenza infection (247). Other in vivo coexposure studies using PM and allergens have shown that prior PM instillation promotes development of a mixed Th2/Th17 phenotype that may perpetuate asthmatic responses (248, 249).

### Epigenetic changes.

Air pollution exposure may affect multiple epigenetic modifications, including alterations in DNA methylation and histone modifications. These changes may result in transient, and potentially permanent, changes in gene expression affecting lung function and causing long-term effects on respiratory health.

Changes in histone modifications due to PM exposure have been demonstrated in vitro (250), in animal models (251), and human studies (252, 253). Relatively less is known about how these changes affect cellular function after exposure to pollution; however, decreased IL-6 secretion from macrophages after long-term in vitro exposure to particles was shown to be associated with altered histone methylation events in the *IL-6* promoter after exposure (241).

Exposure to air pollutants has generally been shown to coincide with global hypomethylation of DNA in various tissues, particularly in DNA repetitive elements. Exposure of rats to traffic-related pollution resulted in hypomethylation of blood and lung tissue LINE-1 elements (251). Hypomethylation of circulating leukocyte LINE-1 elements was shown in humans to correlate with black carbon exposure (254, 255). Total blood cell deoxycytidine methylation and total CpG site methylation was also negatively correlated with residential PM_2.5_ levels (256) and with diesel exhaust exposure in a controlled setting (257). At the gene level, differential methylation of gene elements has been shown to increase or decrease depending on gene and exposure (258, 259). One repeated finding is that exposure to ambient air pollution, diesel exhaust, or secondhand smoke is associated with hypermethylation of the *FOXP3* gene, which leads to suppression of regulatory T cell function and increased asthma severity (260–262).

### Carrier effects.

As discussed above, air pollution can disrupt the airway epithelial barrier, impair mucociliary clearance of pathogens, and impair immune responses, all of which cause greater susceptibility to viral infection; however, air pollution particles themselves are also carriers of virus that can influence viral infectivity. Infective influenza virus can be transmitted between animals on nonrespiratory particles (263). Moreover, a study measuring ambient influenza virus in Taiwan found that ambient virus was significantly higher on days in which air particulates were elevated due to Asian dust storms (264). In a recent study, airborne ambient particles were shown to bind to influenza virus and promote cellular viral uptake in a receptor-independent manner. Furthermore, after nasal instillation, particle-associated virus was taken up deeper into the lung than virus alone, causing greater inflammation and sickness (265).

## Conclusions and future directions

The current evidence shows that air pollution exposure is a major modifiable risk factor for the prevention and management of respiratory disease. As there is no “safe” level of air pollution exposure, efforts to reduce air pollution production will need to be combined with mitigation strategies. Future directions that need to be taken to improve our understanding and to reduce the impact of air pollution on human health are summarized in Table 2. A greater mechanistic understanding of the toxic effects of air pollutants on the lung and other tissues will be required to develop strategies to combat the harmful effects of air pollution exposure. Recent advancements in single-cell transcriptomic and epigenomic techniques will likely play a major role in increasing the understanding of cellular and organismal response to inhaled pollutants. Furthermore, as the associations of air pollution exposure with pulmonary disease become increasingly clear, increased integration of exposure studies with disease modeling studies may help to elucidate the mechanisms by which exposure promotes pulmonary disease.

Advancements in the measurement of air pollutants will be crucial to aid epidemiologic studies by allowing for accurate quantitative assessment of pollutant exposure at greater resolution. While older studies relied on pollutant measurements from the nearest ground-based monitor, often categorizing exposure based on zip code, actual pollutant levels can vary greatly within these areas. Statistical modeling advances such as land use regression analysis and technological advances such as satellite monitoring have increased the resolution of epidemiologic studies; however, these advances still lack fine scale resolution, and data gaps exist due to cloudy days, for example (266). Continued development of low-cost monitor networks may help to increase study resolution and allow for integration of indoor exposures to data sets (267). Finally, studies capable of identifying specific pollutants in the complex mixture that are particularly toxic will also be required to mitigate the effects of air pollution exposure. Analysis of PM elemental composition by the ELAPSE study has shown that V content of particles is most consistently associated with mortality as well as lung cancer incidence (268, 269). Such advancements in epidemiologic analysis may inform future toxicological studies as well as lead to policy changes that limit specific pollutants. These future studies will benefit society as a whole, but they will have an outsized effect on vulnerable populations, including the aged and low-income populations, on whom the effects of reducing air pollution production have been most acutely demonstrated (270, 271).

## Figures and Tables

**Table 2:**
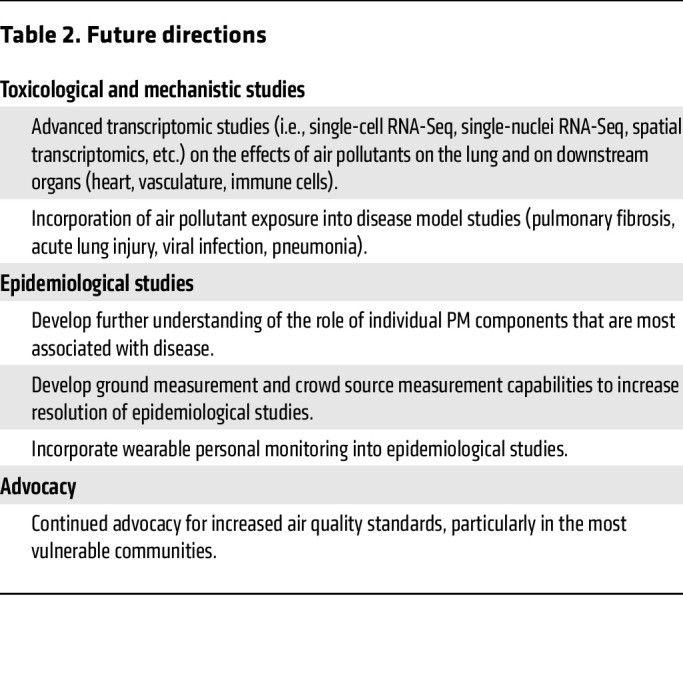
Future directions

**Table 1:**
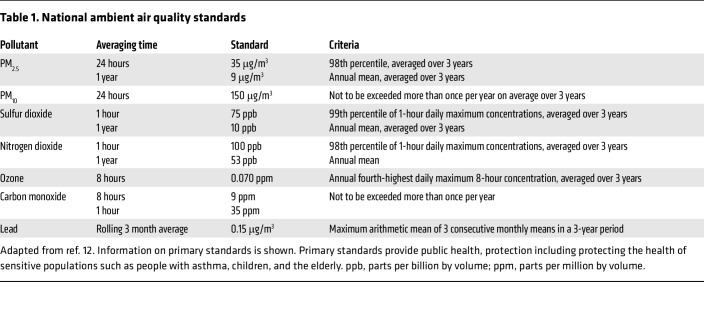
National ambient air quality standards

## Version 1 09/02/2025

Electronic publication
